# Supplementary material for: Explainable Machine Learning to Predict Successful Weaning of Mechanical Ventilation in Critically Ill Patients Requiring Hemodialysis
Source: Healthcare (Basel). 2023 Mar 21;11(6):910. doi: 10.3390/healthcare11060910 (PMC10048210; doi:10.3390/healthcare11060910)
Supplement: Supplementary file 1 [file healthcare-11-00910-s001.zip › healthcare-2275201-supplementary.pdf]

Supplemental Table 1. The applied parameters in the XGBoost, GBM, AdaBoost, and RF.

XGBoost

{'objective': 'binary:logistic', 'use_label_encoder': None, 'base_score': None, 'booster':'gbtree', 'callbacks': None, 'colsample_bylevel': 1, 'colsample_bynode': 1, 'colsample_bytree': 1, 'early_stopping_rounds': None, 'enable_categorical': False, 'eval_metric': 'auc', 'feature_types': None, 'gamma': 0, 'gpu_id': None, 'grow_policy': 'depthwise', 'importance_type': None, 'interaction_constraints': None, 'learning_rate': 0.3, 'max_bin': 256, 'max_cat_threshold': None, 'max_cat_to_onehot': None, 'max_delta_step': 0, 'max_depth': 6, 'max_leaves': 0, 'min_child_weight': 1, 'missing': nan, 'monotone_constraints': None, 'n_estimators': 100, 'n_jobs': None, 'num_parallel_tree': 1, 'predictor': 'auto', 'random_state': None, 'reg_alpha': 0, 'reg_lambda': 0, 'sampling_method': 'uniform', 'scale_pos_weight': 1, 'subsample': 1, 'tree_method': 'auto', 'validate_parameters': 'false', 'verbosity': 1}

GBM

{'ccp_alpha': 0.0, 'criterion': 'friedman_mse', 'init': None, 'learning_rate': 0.1, 'loss': 'log_loss', 'max_depth': 3, 'max_features': None, 'max_leaf_nodes': None, 'min_impurity_decrease': 0.0, 'min_samples_leaf': 1, 'min_samples_split': 2, 'min_weight_fraction_leaf': 0.0, 'n_estimators': 100, 'n_iter_no_change': None, 'random_state': None, 'subsample': 1.0, 'tol': 0.0001, 'validation_fraction': 0.1, 'verbose': 0, 'warm_start': False}

AdaBoost

{'algorithm': 'SAMME.R', 'base_estimator': 'deprecated', 'estimator': None, 'learning_rate': 1.0, 'n_estimators': 50, 'random_state': None}

RF

{'bootstrap': True, 'ccp_alpha': 0.0, 'class_weight': None, 'criterion': 'gini', 'max_depth': None, 'max_features': 'sqrt', 'max_leaf_nodes': None, 'max_samples': None, 'min_impurity_decrease': 0.0, 'min_samples_leaf': 1, 'min_samples_split': 2, 'min_weight_fraction_leaf': 0.0, 'n_estimators': 100, 'n_jobs': None, 'oob_score': False, 'random_state': None, 'verbose': 0, 'warm_start': False}

Abbreviation: Extreme gradient boosting, XGBoost; Gradient Boosting Machine, GBM; Adaptive Boosting, AdaBoost; Random Forest (RF)

Supplemental Table 2. Metrics of performance among distinct machine learning models

	Accuracy[a]	Recall	F1 score	PPV	NPV
**XGBoost**	0.742±0.006	0.741±0.012	0.736±0.006	0.768±0.016	0.732±0.09
**GBM**	0.739±0.018	0.742±0.018	0.728±0.022	0.762±0.012	0.715±0.026
**ADAboost**	0.692±0.014	0.71±0.028	0.691±0.012	0.712±0.035	0.674±0.012
**RF**	0.695±0.011	0.655±0.037	0.669±0.015	0.704±0.024	0.686±0.029

[a] (TP+TN) / (TP+FN+TN+FP). Abbreviation: XGBoost, eXtreme Gradient Boosting; GBM, Gradient Boosting Machine; AdaBoost, Adaptive Boosting; RF, Random Forest.

Supplemental Table 3. Delong test to determine the difference of performance among distinct machine learning models

	**XGBoost**	**GBM**	**ADAboost**	**RF**
**XGBoost**	NA	0.146	<0.01	<0.01
**GBM**	0.146	NA	<0.01	0.011
**ADAboost**	<0.01	<0.01	NA	0.06
**RF**	<0.01	<0.01	0.06	NA

Abbreviation: XGBoost, eXtreme Gradient Boosting; GBM, Gradient Boosting Machine; AdaBoost, Adaptive Boosting; RF, Random Forest.

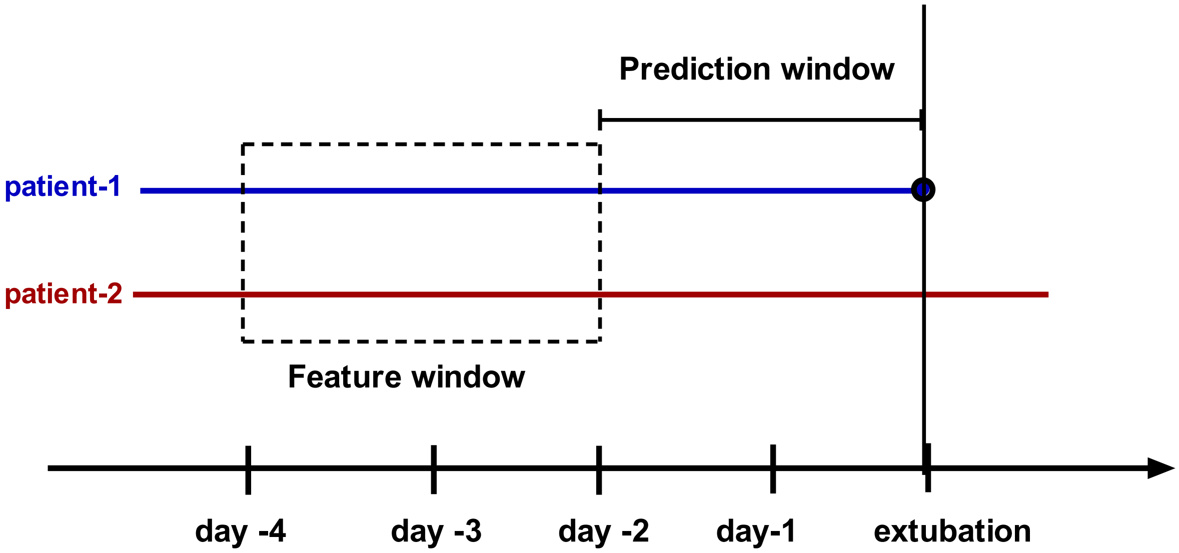

Supplemental Figure 1. Illustration of the study design and the time frame with right alignment. Subjects were aligned at the alignment point that was extubation-day or one random-day in those without extubation. The data within prediction window (day -4 and day -3 prior to extubation-day) were collected, and the prediction window reflects the time of the prediction ahead of extubation.
